# Supplementary material for: Validation of blue- and clear-native polyacrylamide gel electrophoresis protocols to characterize mitochondrial oxidative phosphorylation complexes
Source: PLoS One. 2025 Sep 18;20(9):e0332065. doi: 10.1371/journal.pone.0332065 (PMC12445495; doi:10.1371/journal.pone.0332065)
Supplement: S1 File — Step-by-step protocol, also available on protocols.io, https://doi.org/10.17504/protocols.io.6qpvrkdrolmk/v1. (PDF) [file pone.0332065.s001.pdf]

May 13, 2025

Characterisation of mitochondrial oxidative phosphorylation complexes by blue- and clear-native polyacrylamide gel electrophoresis and in-gel activity staining

DOI
dx.doi.org/10.17504/protocols.io.6qpvrkdrolmk/v1

Jana Aref[1], Seungtae Lee[1], Supachaya Sriphoosanaphan[2], Micol Falabella[3], Jan-Willem Taanman[1]

[1]Department of Clinical and Movement Neurosciences, UCL Queen Square Institute of Neurology, University College London;
[2]Institute for Liver and Digestive Health, University College London;
[3]Department of Neuromuscular Diseases, UCL Queen Square Institute of Neurology, University College London

Jana Aref: Current department: Department of Neuromuscular Diseases, UCL Queen Square Institute of Neurology, University College London;
Seungtae Lee: Current institute: Biodonostia Health Research Institute; 20014 San Sebastián;
Supachaya Sriphoosanaphan: Additional affiliations: Division of Gastroenterology, Department of Medicine, Faculty of Medicine, Chulalongkorn University, Bangkok; Centre of Excellence in Liver Diseases, King Chulalongkorn Memorial Hospital, Thai Red Cross Society, Bangkok

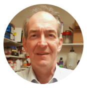

Jan-Willem Taanman
UCL Queen Square Institute of Neurology

OPEN ACCESS

DOI: dx.doi.org/10.17504/protocols.io.6qpvrkdrolmk/v1

Protocol Citation: Jana Aref, Seungtae Lee, Supachaya Sriphoosanaphan, Micol Falabella, Jan-Willem Taanman 2025. Characterisation of mitochondrial oxidative phosphorylation complexes by blue- and clear-native polyacrylamide gel electrophoresis and in-gel activity staining. **protocols.io https://dx.doi.org/10.17504/protocols.io.6qpvrkdrolmk/v1**

License: This is an open access protocol distributed under the terms of the **Creative Commons Attribution License**, which permits unrestricted use, distribution, and reproduction in any medium, provided the original author and source are credited

Protocol status: Working
We use this protocol and it's working

Created: April 09, 2025

Last Modified: May 13, 2025

Protocol Integer ID: 126434

Keywords: Blue native polyacrylamide gel electroph..., Clear native polyacrylamide gel electrophoresis, Electron transport chain, In-gel enzyme activity staining, Mitochondria, Mitochondrial respiratory chain, Oxidative phosphorylation, Western blot analysis

Funders Acknowledgements:
Royal Free Charity
Grant ID: Fund 42

Abstract

The oxidative phosphorylation system plays a pivotal role in the catabolic energy transduction of the cell. The system is composed of five multi-subunit protein complexes that are embedded in the cristae membranes of the cell's mitochondria. Specialised native polyacrylamide gel electrophoresis techniques have been reported to resolve this hydrophobic enzyme system and gain detailed insights into its structure and function. Here, we describe our step-by-step lab protocol that we have developed over the past >20 years to study oxidative phosphorylation defects in tissues and cultured cells derived from patients with severe metabolic disorders. Our protocol includes a description of the sample preparation, pouring of the polyacrylamide gradient gels, one-dimensional native and two-dimensional native/denaturing gel electrophoresis, western blot analysis and in-gel enzyme activity staining. Our protocol is adaptable and yields robust, quantitative and reproducible results.

Guidelines

This protocol is based on the Mini-Protean Tetra Vertical Electrophoresis Cell system from Biorad Laboratories and a Hoeffer four-way gradient mixer connected to a Watson Marlow 205U four-way peristaltic pump.

Suggested timeline

- Read the protocol from beginning to end to familiarise yourself with it.
- Harvest and freeze cell pellets or mitochondrial preps on day 1 or earlier.
- Make up all components for pouring gels, running gels, and western blot analysis that can be stored on day 1 or earlier.
- day 1 Pour four gradient polyacrylamide gels and store at 4°C overnight.
- day 2 Run native polyacrylamide gels and process as required, run and blot second dimension denaturing polyacrylamide gels if required.
- day 2-3 Probe blots with antibodies and develop.

Materials

Specialised equipment

- Four-way peristaltic pump (Watson Marlow, 205S) equipped with yellow-blue PVC pump tubes (0497-4155)
- Four-way gradient mixer, Exponential Gradient Maker; Hoeffer Scientific Instruments, XPO 77
- Mini-PROTEAN Tetra Vertical Electrophoresis Cell for 0.75-mm thick handcast gels; Biorad Laboratories, 1658002FC
- Mini Trans-blot Module; Biorad Laboratories, 1703935
- Mini Cell Buffer Dam; Biorad Laboratories, 1653130
- PowerPac Basic Power Supply; Biorad Laboratories, 1645050
- Chemidoc Imaging System; Biorad Laboratories, 12003153
- Refrigerated microcentrifuge for 1.5-ml reaction tubes

Reagents

- 1-Butanol (*n*-butanol); Sigma-Aldrich/Merck, 537993
- 3-3′-Diaminobenzidine (DAB); Sigma-Aldrich/Merck, D8001
- 6-Aminocaproic acid; Sigma-Aldrich/Merck, A7824
- Acetic acid, glacial; Fisher Scientific, 10744361
- Acetone; Supelco/Merck, 90872
- Acrylamide (powder); Sigma-Aldrich/Merck, A9099
- Acrylamide : *N*-*N*′-methylene-bisacrylamide (37.5% : 1%), 40% (w/v) stock solution; Supelco/Merck, 01709-500ML
- Adenosine 5′-triphosphate disodium salt hydrate (ATP); Sigma-Aldrich/Merck, A2383
- Amberlite MB-1 resin; Sigma-Aldrich/Merck, D2572
- Ammonium persulphate (ammonium peroxodisulphate, APS); VWR Chemicals, 21300.293
- Ammonium sulphide 20% solution, $(NH_4)_2S$, Sigma-Aldrich/Merck, W205303
- Antibodies, primary and secondary, see paragraph 19
- Bis-tris (bis(2-hydroxyethyl)amino tris(hydroxymethyl)methane); VWR Life Sciences, 0715
- Cytochrome-*c* from equine heart; Sigma-Aldrich/Merck, C2867
- Digitonin, 5% (w/v) solution; Thermo Fisher Scientific, BN2006
- Glycerol; Sigma-Aldrich/Merck, G7893
- Glycine; Fisher Scientific, 10467963
- Hydrochloric acid, 37%, HCl; VWR Chemicals, 20252.335
- Lead(II) nitrate, $Pb(NO_3)_2$, Supelco/Merck, 1.07398
- Leupeptin; Roche/Merck, LEU-RO
- Magnesium sulphate hydrate, $MgSO_4.xH_20$; Sigma-Aldrich/Merck, 63139
- NADH disodium salt (reduced β-nicotinamide-adenine dinucleotide); MedChemExpress, HY-F0001
- *N*-dodecyl-β-D-maltoside (DDM; laurylmaltoside); Thermo Fisher Scientific, 89903
- Nitroblue tetrazolium chloride (NBT); Thermo Fisher Scientific, 34035
- *N*-methylphenazonium methylsulfate; Sigma-Aldrich/Merck, 68600
- *N*-*N*′-methylene-bisacrylamide; Sigma-Aldrich/Merck, M7279
- NuPAGE Sample Reducing Agent (10x); Thermo Fisher Scientific, NP0009
- Pepstatin A; Sigma-Aldrich/Merck, P5318

- Phenylmethylsulphonyl fluoride (PMSF); Sigma-Aldrich/Merck, P7626
- Phosphate Buffered Saline 10x (10x PBS, Dulbecco's Phosphate Buffered Saline 10x); Gibco/Fisher Scientific, 14200-067
- Ponceau S; Sigma-Aldrich/Merck, P3504
- Serva blue G (Coomassie Brilliant blue G 250); Serva, 35050
- Skim Milk Powder; Millipore/Merck, 70166-500G
- Sodium deoxycholate (DOC); Sigma-Aldrich/Merck, D6750
- Sodium dihydrogen phosphate dihydrate, $NaH_2PO_4.2H_20$; Supelco/Merck, 1.06342
- Sodium dodecyl sulphate (SDS); Sigma-Aldrich/Merck, L3771
- Sodium hydrogen phosphate dihydrate, $Na_2HPO_4.2H_20$ (disodium hydrogen phosphate dihydrate); Supelco/Merck, 1.06580
- Sodium hydroxide, NaOH; Sigma-Aldrich/Merck, 567530
- Sodium succinate dibasic hexahydrate, $C_4H_4N_2O_4.6H_2O$; Sigma-Aldrich/Merck, S9637
- TEMED (*N*,*N*,*N'*,*N'*-tetramethylethylenediamine); Sigma-Aldrich/Merck, T9281
- Tricine; VWR Life Science, E170
- Tris (Trizma base); Sigma-Aldrich/Merck, T1503
- Triton X-100; Sigma-Aldrich/Merck, X100
- Tween 20; Sigma-Aldrich/Merck, P1379

Other

- 50-ml Screw cap tubes, 114×28 mm; Sarstedt, 62.547.254
- Clarity Western ECL Substrate; Biorad Laboratories, 1705061
- Pierce BCA Protein Assay Kit; Thermo Fisher Scientific, 23225
- Gel Saver II Tip 1-200 µL Starlab, I1022-0610
- Precision Plus Protein Standards; Biorad Laboratories, 161-0374
- PVDF Transfer Membrane 0.2 µm; Thermo Fisher Scientific, 88520
- Whatman 3MM Chromatography Paper; Cytiva, 3030-917
- Transparent overhead sheets

Safety warnings

Biological hazards
The samples being analysed may be from pathological specimens, so precautions should be taken, including the wearing of personal protection equipment (PPE).

Chemical hazards
Many of the reagents are toxic. Wearing of personal protection equipment (PPE) is advised.
Take special care when working with the following toxic reagents:

- Acrylamide
- Ammonium persulphate (APS)
- Ammonium sulphide, $(NH_4)_2S$
- Butanol
- Diaminobenzidine (DAB)
- Digitonin
- Methanol
- N-dodecyl-β-D-maltoside powder (DDM)
- *N*,*N*,*N*′,*N*′-tetramethylethylenediamine (TEMED)
- Lead (II)nitrate, $Pb(NO_3)_2$
- Phenylmethylsulphonyl fluoride (PMSF)
- Sodium dodecylsulphate powder (SDS)
- Sodium deoxycholate powder (DOC)

Electrical hazards
Ensure the power pack and cables are not damaged and work correctly.

Fire hazards
Do not leave electrical equipment running unattended for long periods. Check it regularly.
Acetone, butanol and methanol are highly flammable.

Ethics statement

Ethics approval from the local Ethics Committee is required if human samples are studied.
Experiments involving animals must be conducted according to internationally-accepted standards and should have prior approval from an Institutional Animal Care and Use Committee or equivalent ethics committee(s).

Pouring of gradient gels

1 **Components**

Acrylamide-bis (AB): 48% acrylamide, 1.5% bisacrylamide (49.5%T 3%C)

- 48 g of acrylamide, 1.5 g of *N-N'*-methylene-bisacrylamide made up to 100 ml with ddH_2O.
- Add some Amberlite MB resin for trace contaminant removal if required (i.e., if the solution is not colourless; 1-3 h stirring at room temperature).
- Filter through filter unit.
- Store at 4°C in the dark.

3x gel buffer (3X): 1.5 M 6-aminocaproic acid, 150 mM bis-tris.

- 39.36 g of 6-aminocaproic acid, 6.28 g of bis-tris, pH 7.0 with HCl and make up to 200 ml with ddH_2O.
- Store at 4°C.

10% Ammonium persulphate (APS)

- 10% (w/v) ammonium persulphate in ddH_2O. For instance, 100 mg of ammonium persulphate in 1000 µl of ddH_2O.
- Will keep for ~1 month when stored at 4°C.

Water-saturated butanol

- Mix equal volumes of ddH_2O and n-butanol in a small bottle, shake and leave to settle.
- Butanol is the top layer.
- Store at room temperature.

- **TEMED** (*N,N,N',N'*-tetramethylethylenediamine)
- Store at room temperature.

Glycerol

- Store at room temperature.

Gel loading tips

- Gel Saver II Tip 1-200 µL; Starlab, Cat. No.: I1022-0610

2 **Gel solutions**

Choose the appropriate gel gradient for your application. A 3-10% separating gel with a 3% stacking gel gives good resolution of Complex I, III and IV supercomplexes. A 3-12%

separating gel with a 3% stacking gel gives good resolution of the five oxidative phosphorylation complexes. A 8-16% separating gel with a 4% stacking gel gives good resolution of Complex IV subassemblies.

Table 1 **Low percentages for separating gel**

Reagent	3%	4%	5%	8%	10%
AB	0.744 ml	0.99 ml	1.24 ml	1.98 ml	2.48 ml
3X	3.98 ml	3.98 ml	3.98 ml	3.98 ml	3.98 ml
ddH_2O	7.10 ml	6.85 ml	6.60 ml	5.87 ml	5.38 ml
10% APS	70 µl	70 µl	70 µl	60 µl	50 µl
TEMED	10 µl	10 µl	9 µl	8 µl	7 µl

Table 2 **High percentages for separating gel**

Reagent	8%	10%	12%	13%	16%	18%
AB	1.63 ml	2.02 ml	2.44 ml	2.64 ml	3.25 ml	3.63 ml
3X	3.30 ml	3.30 ml	3.30 ml	3.30 ml	3.30 ml	3.30 ml
ddH_2O	3.32ml	2.93 ml	2.51 ml	2.31 ml	1.70 ml	1.32 ml
Glycerol	1.98 g	1.98 g	1.98 g	1.98 g	1.98 g	1.98 g
10% APS	35 µl	35 µl	35 µl	35 µl	35 µl	35 µl
TEMED	5 µl	5 µl	5 µl	5 µl	5 µl	5 µl

Table 3 **Stacking gel**

Reagent	3%	4%
AB	0.38 ml	0.50 ml
3X	2.00 ml	2.00 ml
ddH_2O	3.12 ml	3.50 ml
10% APS	50 µl	50 µl
TEMED	10 µl	10 µl

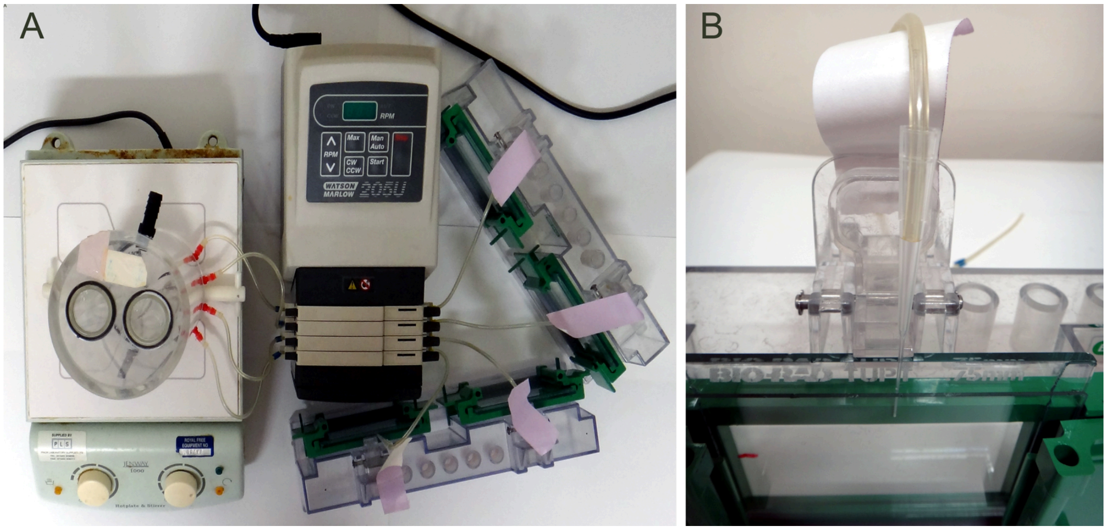

Figure 1 Pouring of four gradient gels with a four-way gradient mixer (on a magnetic stirrer) connected to a four-way peristaltic pump. (**A**) Setup (steps 3.1-6), (**B**) Gel loading tip mounted onto the end of a tube and with its tip between a pair of glass plates (step 3.6).

3 Protocol

For pouring four 0.75-mm thick gradient gels with the Mini-Protean Tetra Vertical Electrophoresis Cell system (Biorad Laboratories) with a four-way gradient mixer (Hoeffer) connected to a four-way peristaltic pump (Watson Marlow).

3.1 Ensure all equipment is clean and free from dust.

3.2 Assemble glass plates with a 0.75-mm spacer as per BioRad protocol.

3.3 Connect the four exit taps of the gradient mixer to the four tubes of the peristaltic pump (Fig. 1A).

3.4 Place the gradient mixer on a magnetic stirrer and place a small magnetic stir bar in each of the cylinders of the gradient mixer.

3.5 Ensure that the tap allowing liquid into the front chamber (with the four exit taps) is closed and open all other taps.

3.6 Push a gel loading tip tightly onto the end of each of the four tubes exiting the pump and push the tips into the top of each of the four pairs of glass plates (Fig. 1B). Secure tubes

with tape.

3.7 Make up a high percentage and a low percentage gel solution in 25-ml beakers (Table 1 and 2), ensuring that the high percentage gel is stirred with a magnetic stir bar as the glycerol is very viscous. Add the TEMED and APS at the end and mix.

3.8 Pipette 7.2 ml of the high percentage gel solution into the front chamber (with the four exit taps) and 7.2 ml of the low percentage gel solution into the back chamber.

3.9 Start stirring the gels solutions at a medium speed in the gradient mixer.

3.10 Turn on the pump at a speed of 1.0 ml/min/tube and allow the high percentage gel solution to flow ~2 cm towards the pump.

3.11 Open tap allowing the low percentage gel solution from the back chamber into the front chamber to start creating a gradient. There is sometimes an air bubble in the channel between the two chambers that obstructs the flow. Therefore, apply some pressure by putting the palm of your hand on the top of the back chamber.

3.12 Resist any temptation to interfere with the pouring at this stage. Never turn off a tap to a gel if it starts to leak, simply put the tube running to it into a waste beaker.

3.13 Once poured, immediately remove the tips/tubes from the glass plates and rinse the gradient mixer and tubes through by pumping a large volume of ddH_2O at maximum speed into a waste beaker.

3.14 Overlay the gels with 200 µl of water-saturated butanol and leave to polymerise. This will take 10-30 min depending on the ambient temperature and concentration of APS and TEMED.

3.15 Make up a stacking gel solution in a 25-ml beaker without adding the APS or TEMED (Table 3).

3.16 When the gradient gels have polymerised, carefully remove the butanol and the layer of water (from the polymerisation) from the top of the gels by upturning them over a sink and mop up the excess with filter paper.

3.17 Add the APS and TEMED to the stacking gel solution and mix.

3.18 Pour the stacking gel and insert the appropriate comb. Make sure there are no air bubbles trapped under the teeth of the comb.

3.19 Leave the stacking gel to polymerise. Gels should be left >45 min after the stacking gel has set before use. Alternatively, once the gels have set they can be wrapped in cling film and stored upright for up to 3 days at 4°C.

Sample preparation of cell culture or mitochondrial pellets for resolution of the five oxidative phosphorylation complexes

4 Use cell culture pellets derived from >1 × 10^6 cells or use mitochondrial pellets derived from >5 mg of tissue.

All steps should be carried out on ice. For western blot analysis of extracts from cultured cells use ≥10 µg of protein per lane, for mitochondria use <10 µg of protein per lane. Use 20–30 µg for in-gel activity staining of cell culture extracts. Use ~10 µg for in-gel activity staining of mitochondrial samples. As the Complex V in-gel activity stain is quite sensitive, 50% less protein can be used. Optimal amounts of protein will depend on cell or tissue type.

The extract in 0.5% n-dodecyl-β-D-maltoside (DDM) can be used for blue-native and clear-native polyacrylamide gels, but also for spectrophotometric or plate reader enzymatic assays of e.g., cytochrome-*c* oxidase and citrate synthase activity.

Components

2x Protein solubilising solution (2x PSS): 2 M 6-aminocaproic acid, 100 mM bis-tris (pH7.0)
- 5.248 g of 6-aminocaproic acid, 0.420 g of bis-tris. pH 7.0 with HCl and make up to 20 ml with ddH_2O.
- Store 4°C.

N-dodecyl-β-D-maltoside (DDM): 20% (w/v) solution of n-dodecyl-β-D-maltoside
- 2 g in 10 ml of ddH_2O to make 20%. (Dissolve in 12-ml tube on bottle roller).
- Store at 4°C.

Protease inhibitors (Alternatively, a commercially available protease inhibitor cocktail can be used)
Phenylmethylsulfonyl fluoride (PMSF): 1 M PMSF in acetone.
- 174 mg/ml of acetone.
- Store at -20°C.

Leupeptin

- 1 mg/ml of ddH_2O.
- Store at -20°C.

Pepstatin A
- 1 mg/ml of methanol.
- Store at -20°C.

Loading buffer (LB) for blue-native gels: 1 M 6-aminocaproic acid, 5% Serva blue G
- 1.312 g of 6-aminocaproic acid, 0.5 g of Serva blue G. Make up to 10 ml with ddH_2O in a 12-ml tube (no need to pH).
- Rotate overnight on a bottle roller to dissolve Serva blue G.
- Store at 4°C.

50% (v/v) glycerol (for clear native gels)
- Mix 1:1 (v/v) glycerol and ddH_2O.
- Store at room temperature.

0.1% (w/v) Ponceau S (for clear native gels)
- 0.01 g of Ponceau S in 10 ml of ddH_2O.
- Store at 4˚C.

Pierce BCA Protein Assay Kit, Thermo Scientific, Cat. No.: 23225

5 **Protocol**

Everything on ice; pre-cool micro-centrifuge to 4°C.

5.1 Extract samples (cell culture pellets, mitochondrial pellets) in 1x PSS, 0.5% DDM, 2 mM PMSF, 2 µg/ml leupeptin and 2 µg/ml of pepstatin A (DDM extraction solution).

Make up sufficient DDM extraction solution for all samples. Use 100 µl for small pellets (1 x 10^6 cells or mitochondria isolated from 5 mg of tissue), use a larger volume for larger pellets.

For instance, to make 1 ml of DDM extraction solution, mix:
- 469 µl of ddH_20
- 500 µl of 2x PSS
- 25 µl of 20% DDM (Check that DDM is fully dissolved after storage at 4˚C; if not, warm up to dissolve)
- 2 µl 1 M PMSF stock
- 2 µl of 1 mg/ml of leupeptin stock

- 2 µl of 1 mg/ml of pepstatin A stock

5.2 Extract samples (cell culture pellets, mitochondrial pellets) in a reaction tube by adding the appropriate volume of DDM extraction solution and pipetting up and down to resuspend the pellet. Vortex samples briefly and keep on ice for 15 min. Vortex every 5 min.

5.3 After 15 min on ice, microfuge at maximum speed (16,000 x *g*) for 20 min at 4°C.

5.4 Transfer supernatant to a new labelled tube on ice.

5.5 Carry out a protein assay to determine the protein concentration, e.g. the BCA protein assay in quadruplicate. A sample volume of 2 µl is normally sufficient per single assay (i.e., 4 × 2 µl for quadruplicate assays).

5.6 Decide which volume of the samples you want to use for blue-native gel electrophoresis, which volume for clear-native gel electrophoresis and which volume for spectrophotometric/plate reader assays of e.g., cytochrome-c oxidase and citrate synthase.

Preparation of samples for blue-native polyacrylamide gels (western blotting of Complexes I, II, III and IV, and in-gel activity of Complex I)
Using the results of the protein assay, adjust the volume of the various samples to an equal protein concentration with DDM extraction buffer. Then add 1/6 of a volume of loading buffer (LB) and mix.

Preparation of samples for clear-native polyacrylamide gels (western blotting of Complex V and in-gel activity of Complexes II, IV and V)
Using the results of the protein assay, adjust the volume of the various samples to an equal protein concentration with DDM extraction buffer. Then add 1/10 of a volume of 50% glycerol and 1/10 of a volume of 0.1% Ponceau S and mix.

Samples can be stored at -70˚C for several weeks.

Sample preparation of cell culture or mitochondrial pellets for resolution of Complex I-III-IV supercomplexes

6 **Components**

2x Protein solubilising solution (2x PSS): 2 M 6-aminocaproic acid, 100 mM bis-tris (pH7.0)

- 5.248 g of 6-aminocaproic acid, 0.420 g of bis-tris. pH 7.0 with HCl and make up to 20 ml with ddH_2O.
- Store 4°C.

Digitonin: 5% (w/v) solution of digitonin
- ThermoFisher Scientific, Cat. No.: 2006.
- Store at 4°C.

Protease inhibitors (Alternatively, a commercially available protease inhibitor cocktail can be used)
Phenylmethylsulfonyl fluoride (PMSF): 1 M PMSF in acetone.
- 174 mg/ml of acetone.
- Store at -20°C.

Leupeptin
- 1 mg/ml of ddH_2O.
- Store at -20°C.

Pepstatin A
- 1 mg/ml of methanol.
- Store at -20°C.

Loading buffer (LB) for blue-native gels: 1 M 6-aminocaproic acid, 5% Serva blue G
- 1.312 g of 6-aminocaproic acid, 0.5 g of Serva blue G. Make up to 10 ml with ddH_2O in a 12-ml tube (no need to pH).
- Rotate overnight on a bottle roller to dissolve Serva blue G and store at 4°C.

Pierce BCA Protein Assay Kit, Thermo Scientific, Cat. No.: 23225

7 **Protocol**

Everything on ice; pre-cool micro-centrifuge to 4°C.

7.1 Extract samples (cell culture pellets, mitochondrial pellets) in 1x PSS, 2% digitonin, 2 mM PMSF, 2 µg/ml leupeptin and 2 µg/ml of pepstatin A (digitonin extraction solution).

Make up sufficient digitonin extraction solution for all samples. Use 100 µl for small pellets (1 × 10^6 cells or mitochondria isolated from 5 mg of tissue), use a larger volume for larger pellets.

For instance, to make 1 ml of digitonin extraction solution, mix:
- 94 µl of ddH_20

- 500 µl of 2x PSS
- 400 µl of 5% digitonin (If the digitonin is precipitated, heat the solution at 95°C for 5 minutes or until fully dissolved, then cool to room temperature).
- 2 µl 1 M PMSF stock
- 2 µl of 1 mg/ml of Leupeptin stock
- 2 µl of 1 mg/ml of Pepstatin A stock

7.2 Extract samples (cell culture pellets, mitochondrial pellets) by adding the appropriate volume of digitonin extraction solution and pipetting up and down to resuspend the pellet. Vortex samples briefly and keep on ice for 15 min. Vortex every 5 min.

7.3 After 15 min on ice, microfuge at maximum speed (16,000 x *g*) for 15 min at 4°C.

7.4 Transfer supernatant to a new labelled tube on ice.

7.5 Carry out a protein assay to determine the protein concentration, e.g. the BCA protein assay, in quadruplicate. A sample volume of 2 µl is normally sufficient per single assay (i.e., 4 × 2 µl for quadruplicate assays).

7.6 Using the results of the protein assay, adjust the volume of the various samples to an equal protein concentration with digitonin extraction buffer.

Preparation of samples for blue-native polyacrylamide gels (western blotting and in-gel activity of Complexes I-III-IV supercomplexes)
Using the results of the protein assay, adjust the volume of the various samples to an equal protein concentration with digitonin extraction buffer. Then add 1/6 of a volume of loading buffer (LB) and mix.

Samples can be stored at -70˚C for several weeks.

Running blue-native and clear-native gels

8 **Components**

Anode buffer (AB): 50 mM bis-tris
For all blue- and clear-native gel electrophoresis
- 10.46 g of bis-tris, pH to 7.0 with HCl, make up to 1 L with ddH_2O.
- Store at 4˚C.

Cathode buffer A (CBa): 50 mM tricine, 15 mM bis-tris, 0.02% Serva blue G

For blue-native gel electrophoresis to perform western blot analyses of Complex I, II, III and IV, and Complex I-III-IV supercomplexes.

- 8.96 g of tricine, 3.13 g of bis-tris, 200 mg of Serva blue G. pH to 7.0 with HCl/NaOH and make up to 1 L with ddH_2O.
- To dissolve the blue dye, stir overnight at room temperature.
- Store at 4°C.

Cathode buffer B (CBb): 50 mM tricine, 15 mM bis-tris (as above but without Serva blue G)
For blue-native gel electrophoresis to perform in-gel Complex I and in-gel Complex I-III-IV supercomplex activity staining.

- 8.96 g of tricine, 3.13 g of bis-tris. pH to 7.0 with HCl/NaOH and make up to 1 L with ddH_2O.
- Store at 4°C.

Cathode buffer C (CBc): 50 mM tricine, 15 mM bis-tris, 0.05% (v/v) Triton X-100, 0.05% (w/v) DOC
For clear-native gel electrophoresis to perform in-gel activity staining of Complex IV, and western blot analysis and in-gel activity staining of complex V.

- 8.96 g of tricine, 3.13 g of bis-tris. pH to 7.0 with HCl/NaOH and make up to 1 L with ddH_2O.
- Add 0.5 ml of Triton X-100.
- Add 0.5 g of sodium deoxycholate (DOC).
- Stir until fully dissolved.
- Store at 4°C.

Cathode buffer D (CBd): 50 mM tricine, 15 mM bis-tris, 0.02% (w/v) DDM, 0.05% (w/v) DOC
For clear-native gel electrophoresis to perform in-gel activity staining of Complex II.

- 8.96 g of tricine, 3.13 g of bis-tris. pH to 7.0 with HCl/NaOH and make up to 1 L with ddH_2O.
- Add 0.2 g of *n*-dodecyl-β-D-maltoside (DDM)
- Add 0.5 g of sodium deoxycholate (DOC).
- Stir until dissolved.
- Store at 4°C.

9 **Protocol**

For running a single gel with a Mini Cell Buffer Dam in place of the companion gel or for running one pair of gels with the Mini-Protean Tetra Vertical Electrophoresis Cell system (Biorad Laboratories).

9.1 Assemble the gels in the Mini-protean gel holder as directed by Biorad Laboratories, taking care to remove any gel residue from outside of plates (rinse with tap water) that might interfere with seal around the buffer chambers.
Remove the combs and straighten the wells with a micro-spatula if necessary.
If running blue-native gels, do not put the gels in the electrophoresis tank yet; it is easier to load the gels when not in the tank.
For blue-native gels, illuminate gels from behind to increase visibility of the wells and facilitate loading.

9.2 Fill the inner chamber with CBa, CBc or CBd depending on the experiment.

9.3 Rinse out wells of the first gel, using a syringe filled with CBa, CBc or CBd and equipped with a gel loading tip.

9.4 Load the first gel and then rinse wells from the second gel and load.

9.5 Put the gels in the electrophoresis tank and pour ~330 ml of AB into the tank to ~2 cm above the bottom of the glass plates.

9.6 *For western blot analysis of Complexes I, II, III and IV, and Complex I-III-IV supercomplexes*

- Run gels at 100 V until front is well into stacking gel (15 min).
- Then run at 4-5 mA fixed current per gel (i.e. 8-10 mA for two gels) until the blue front runs off.
- For Complex I-III-IV supercomplexes, run for another 30 min after the blue front starts to run off.

For in-gel Complex I and Complex I-III-IV supercomplex activity staining

- Run gels at 100 V until front is well into stacking gel (15 min).
- Then change buffer in inner chamber to CBb and run at 4-5 mA fixed current per gel (i.e. 8-10 mA for two gels) until the blue front runs off. (Changing of the buffer allows better visualisation of the bands).
- For Complex I-III-IV supercomplexes, run for another 30 min after the blue front starts to run off.

For in-gel Complex II, IV or V activity staining or Complex V western blot analysis

- Run gels at 100 V until front is well into stacking gel (15 min).
- Then run at 4-5 mA fixed current per gel (i.e. 8-10 mA for two gels) until the red front runs off.

9.7 Process gels as required.

In-gel enzyme activity staining

10 **NADH dehydrogenase stain (Complex I)**

Use a blue-native gel which was run with cathode buffer containing 0.02% Serva blue G (CBa) for 15 min, followed by running with the cathode buffer without dye (CBb).

Stain solution for 1 gel
30 ml of 2 mM Tris·HCl (pH 7.4), 0.1 mg/ml of NADH, 2.5 mg/ml of nitroblue tetrazolium.

- Prepare a 1 M Tris·HCl (pH 7.4) stock solution (store at room temperature).
- Weight out 3 mg of NADH.
- Weight out 75 mg of nitroblue tetrazolium.
- Shortly before use, dissolve NADH and nitroblue tetrazolium in 30 ml of ddH_2O in a 50-ml screw top tube by vortexing.
- Add 60 µl 1 M Tris·HCl (pH 7.4) and mix.

Stop solution: 40% (v/v) methanol, 10% (v/v) acetic acid

- Mix 250 ml of ddH_2O, 200 ml of methanol and 50 ml of glacial acetic acid to make 0.5 L of stop solution.
- Store at room temperature.

10.1 Make up stain solution shortly before use.

10.2 When electrophoresis has completed, carefully remove the gel from the glass plates by placing a single-blade razor blade between the glass plates to separate the glass plates. Then cut with the razor blade along the spacers to ensure that the gel does not rip when it is removed. Lift the gel from the bottom with tweezers/gloved fingertips, as this is the strongest part.

10.3 Place the gel in a suitable container, e.g. the plastic lid of a pipet tip box.

10.4 Rinse briefly in ddH_2O.

10.5 Incubate the gel in freshly made staining solution at 37°C, whilst slowly shaking for 1–3 h to develop (~1 h for mitochondrial samples). Cover container, e.g. with a glass plate, to prevent evaporation. Check regularly to avoid overstaining.

10.6 Once the violet staining near the top of the gel has reached a desired intensity, stop reaction and fix the gel in 10% (v/v) acetic acid, 40% (v/v) methanol stop solution for >30 min. This will also destain some residual Serva Blue G. After fixation, replace with ddH_2O.

10.7 After several hours or overnight incubation with ddH_2O, make a photographic record of gel on a light box.

10.8 Quantify bands with ImageJ software.

11 **Succinate dehydrogenase stain (Complex II)**

Use a clear-native gel ran with cathode buffer D (CBd).

Stain solution for 1 gel
30 ml of 2 mM Tris·HCl (pH 7.4), 84 mM sodium succinate, 0.2 mM *N*-methylphenazonium methylsulfate, 2.5 mg/ml of nitroblue tetrazolium.

- Prepare a 1 M Tris·HCl (pH 7.4) stock solution (store at room temperature).
- Weight out 680 mg of sodium succinate ($C_4H_4Na_2O_4.6H_2O$).
- Weight out 75 mg of nitroblue tetrazolium.
- Weight out 12 mg of *N*-methylphenazonium methylsulfate.
- Shortly before use, dissolve sodium succinate, nitroblue tetrazolium and N-methylphenazonium methylsulfate in 30 ml of ddH_2O in a 50-ml screw top tube by vortexing.
- Add 60 µl 1 M Tris·HCl (pH 7.4) and mix.

Stop solution: 40% (v/v) methanol, 10% (v/v) acetic acid

- Mix 250 ml of ddH_2O, 200 ml of methanol and 50 ml of glacial acetic acid to make 0.5 L of stop solution.
- Store at room temperature.

11.1 Make up stain solution shortly before use.

11.2 When electrophoresis has completed, carefully remove the gel from the glass plates by placing a single-blade razor blade between the glass plates to separate the glass plates. Then cut with the razor blade along the spacers to ensure that the gel does not rip when it is removed. Lift the gel from the bottom with tweezers/gloved fingertips, as this is the strongest part.

11.3 Place the gel in a suitable container, e.g. the plastic lid of a pipet tip box.

11.4 Rinse briefly in ddH_2O.

11.5 Incubate the gel in freshly made staining solution at 37°C, whilst slowly shaking for 2–3 h to develop (~2 h for mitochondrial samples). Cover container, e.g. with a glass plate, to prevent evaporation. Check regularly to avoid overstaining.

11.6 Once the violet staining near the bottom of the gel has reached a desired intensity, stop reaction and fix the gel in 10% (v/v) acetic acid, 40% (v/v) methanol stop solution for >30 min. After fixation, replace with ddH_2O.

11.7 After several hours or overnight incubation with ddH_2O, make a photographic record of gel on light box.

11.8 Quantify bands with ImageJ software.

12 **Cytochrome-*c* oxidase stain (Complex IV)**

This stain is often too insensitive to reveal Complex IV activity in samples from whole cell extracts, but it works well with mitochondrial samples after a long incubation in stain solution.

Use a clear-native gel ran with cathode buffer C (CBc) for Complex IV staining or use a blue-native gel ran with CBa for 15 min, followed by CBb for Complex I-III-IV supercomplex staining.

Stain solution for 1 gel
30 ml 50 mM sodium phosphate buffer (pH 7.4), 0.5 mg/ml of 3,3'-diaminobenzidine, 1 mg/ml of equine heart cytochrome-*c*

- Prepare a 50 mM sodium phosphate buffer (pH 7.4) as follows: Dissolve 0.445 g of $Na_2HPO_4.2H_20$ in 50 ml of ddH_2O and dissolve 0.390 g of $NaH_2PO_4.2H_20$ in 50 ml. Then, pipet slowly the NaH_2PO_4 solution (~15 ml) to the 50 ml of Na_2HPO_4 solution in a 100-ml beaker with a pH meter electrode, whilst stirring with a magnetic stir bar until pH 7.4 is reached. This 50 mM sodium phosphate buffer (pH 7.4) can be stored for short periods at 4°C or for longer periods at -20°C .
- Weight out 15 mg of 3,3'-diaminobenzidine (DAB).
- Weight out 30 mg of equine heart cytochrome-*c*.
- Shortly before use, dissolve diaminobenzidine and equine heart cytochrome-*c* separately in ~4 ml of 50 mM sodium phosphate buffer (pH 7.4) by vortexing. Diaminobenzidine requires extensive vortexing to dissolve. Even then, it may not fully

dissolve but it can still be used when partly dissolved. Pool diaminobenzidine and cytochrome-*c* solutions and bring volume to 30 ml with sodium phosphate buffer.

Stop solution: 40% (v/v) methanol, 10% (v/v) acetic acid

- Mix 250 ml of ddH_2O, 200 ml of methanol and 50 ml of glacial acetic acid to make 0.5 L of stop solution.
- Store at room temperature.

12.1 Make up stain solution shortly before use.

12.2 When electrophoresis has completed, carefully remove the gel from the glass plates by placing a single-blade razor blade between the glass plates to separate the glass plates. Then cut with the razor blade along the spacers to ensure that the gel does not rip when it is removed. Lift the gel from the bottom with tweezers/gloved fingertips, as this is the strongest part.

12.3 Place the gel in a suitable container, e.g. the plastic lid of a pipet tip box.

12.4 Rinse briefly in ddH_2O.

12.5 Incubate the gel in freshly made staining solution at 37°C, whilst slowly shaking for 12-16 h to develop. Cover container, e.g. with a glass plate, to prevent evaporation. Check regularly to avoid overstaining.

12.6 Once the brown staining in the middle of the gel has reached a desired intensity, stop reaction and fix the gel in 10% (v/v) acetic acid, 40% (v/v) methanol stop solution for >30 min. After fixation, replace with ddH_2O.

12.7 After several hours or overnight incubation with ddH_2O, make a photographic record of gel on light box.

12.8 Quantify bands with ImageJ software.

13 **ATPase stain (Complex V)**

Use a clear-native gel ran with cathode buffer C (CBc).

Stain solution for 1 gel

30 ml 34 mM Tris·HCl, 270 mM glycine, 8 mM ATP, 14 mM $MgSO_4$, 0.2% (w/v) $Pb(NO_3)_2$ (pH7.4)

- Weight out 120 mg of Tris.
- Weight out 246 mg of glycine.
- Weight out 132 mg of ATP.
- Weight out 50 mg of $MgSO_4$ hydrate.
- Weight out 60 mg of $Pb(NO_3)_2$.
- Shortly before use, dissolve Tris, glycine, ATP, $MgSO_4$ and $Pb(NO_3)_2$ in 30 ml of ddH_2O by vortexing. This solution should have a pH ~7.4. The solution will remain cloudy.

Stain enhancer solution for 1 gel
30 ml of 1.5% (w/v) $(NH_4)_2S$ (ammonium sulphide)

- In a hood (!), dilute 1.5 ml of 20% (w/v) $(NH_4)_2S$ with 28.5 ml of ddH_2O shortly before use.

(Optional) Fix solution: 40% (v/v) methanol

- Mix 300 ml of ddH_2O and 200 ml of methanol to make 0.5 L of fix solution. Do not use acetic acid because the stain will dissolve and disappear under acidic conditions!
- Store at room temperature.

13.1 Make up stain solution shortly before use.

13.2 When electrophoresis has completed, carefully remove the gel from the glass plates by placing a single-blade razor blade between the glass plates to separate the glass plates. Then cut with the razor blade along the spacers to ensure that the gel does not rip when it is removed. Lift the gel from the bottom with tweezers/gloved fingertips, as this is the strongest part.

13.3 Place the gel in a suitable container, e.g. the plastic lid of a pipet tip box.

13.4 Rinse briefly in ddH_2O.

13.5 Incubate the gel in freshly made stain solution at 37°C, whilst slowly shaking for 1-2 h to develop (1 h for mitochondrial samples). Cover container, e.g. with a glass plate, to prevent evaporation. Check regularly for a white precipitate band in the upper half of the gel. Continue with the next step when a white precipitate band is observed. If after 2 h still no white precipitate band is observed, then also continue with the next step. The

following stain enhancement will probably still visualise the Complex V activity, even if no white precipitate band is observed.

13.6 Rinse the gel twice with >100 ml of ddH_2O.

13.7 In a fume hood (!), add the 1.5% (w/v) $(NH_4)_2S$ stain enhancer solution and shake the solution gently to cover the entire gel quickly. Dark brown stain will develop within seconds. Have ddH_2O ready and, after ~10 s in stain enhancer, rinse gel several times with ddH_2O to stop the staining reaction.

13.8 The staining can be fixed in 40% (v/v) methanol solution for >30 min but this step is optional. After fixation, replace with ddH_2O.

13.9 Stained bands will disappear completely within days. Therefore, make a photographic record of gel on light box soon after staining. Often a black precipitate is present on the gel after the treatment with the stain enhancer solution. This can be removed from both sides of the gel by very carefully pulling thick tissue paper over the gel surface until the precipitate is removed.

13.10 Quantify bands with ImageJ software.

Second dimension denaturing polyacrylamide gel electrophoresis

14 This protocol describes the use of SDS-denaturing polyacrylamide gels to resolve the protein complexes separated by native gel electrophoresis under denaturing conditions. This protocol is designed for the use of up to four 0.75-mm thick gels with the Mini-Protean Tetra Vertical Electrophoresis Cell system from Biorad Laboratories.

Components

40% (w/v) Acrylamide : *N*-*N'*-methylene-bisacrylamide (37.5% : 1%) stock solution

- Supelco/Merck, Cat. No.: 01709-500ML
- Store at 4°C in the dark.

10% Ammonium persulphate (APS)

- 10% (w/v) ammonium persulphate in ddH_2O. For instance, 100 mg of ammonium persulphate in 1000 µl of ddH_2O.
- Will keep for ~1 month when stored at 4°C.

10x Reducing agent

- NuPAGE Sample Reducing Agent (10x); Thermo Fisher Scientific, Cat. No.: NP0009

- Store at 4°C.

10% SDS stock solution:

- 10% (w/v) sodium dodecylsulphate in ddH_2O. For instance, dissolve 10 g of sodium dodecyl sulphate (SDS) in 100 ml of ddH^2O.
- Store at room temperature.

1 M Tris.HCl (pH 6.8) stock solution

- 30.28 g of Tris, pH 6.8 with HCl and make up to 250 ml with ddH_2O.
- Store at room temperature.

1.5 M Tris.HCl (pH 8.6) stock solution

- 45.41 g of Tris, pH 8.6 with HCl and make up to 250 ml with ddH_2O.
- Store at room temperature.

TEMED

- Store at room temperature.

Water-saturated butanol

- Mix equal volumes of ddH_2O and 1-butanol (*n*-butanol) in a small bottle, shake and leave to settle.
- Butanol is the top layer.
- Store at room temperature.

Reducing buffer (RB): 20 ml of 0.1x reducing agent, 1% (w:v) SDS

- Pipette 0.2 ml of 10x reducing agent and 2 ml of 10% SDS stock solution in a 50-ml tube and bring to a volume of 20 ml with ddH_2O.
- Make fresh.

Equilibrating buffer (EB): 50 mM Tris.HCl pH 6.8, 1% (w:v) SDS

- Pipette 2.5 ml of 1.0 M Tris.HCl (pH 6.8) stock solution and 5 ml of 10% SDS stock solution in a 50-ml tube and bring to a volume of 50 ml with ddH_2O.
- Can be stored for a short period (weeks) at room temperature.

Polyacrylamide gel running buffer (PB): 25 mM Tris, 192 mM glycine, 0.1% (w/v) SDS

- 14.4 g of glycine, 3 g of Tris, 1 g of SDS made up to 1 L in ddH_2O.
- Store at room temperature.

Precision Plus Protein Standards

- Biorad Laboratories, Cat. No.: 161-0374.
- Store at -20°C.

15 **Gel solutions**

Choose the appropriate gel percentage for your application: 10% for resolution of 20-80 kDa proteins, 12% for resolution of 12-60 kDa proteins and 15% for resolution of 10-43 kDa proteins.

Table 4 **Separating gel**

Reagent	10%	12%	15%
ddH_2O	9.6 ml	8.6 ml	7.1 ml
1.5 M Tris.HCl (pH 8.6)	5.0 ml	5.0 ml	5.0 ml
40% acrylamide : bis (37.5 : 1)	5.0 ml	6.0 ml	7.5 ml
10% SDS	200 µl	200 µl	200 µl
10% APS	200 µl	200 µl	200 µl
TEMED	12 µl	12 µl	12 µl

Table 5 **Stacking gel**

Reagent	5%
ddH_2O	4.35 ml
1 M Tris.HCl (pH 6.8)	750 µl
40% acrylamide : bis (37.5 : 1)	750 µl
10% SDS	60 µl
10% APS	60 µl
TEMED	10 µl

16 **Protocol**

16.1 Cut out single lanes with a resolved sample from the native gel with a single-blade razor blade.

16.2 Soak each gel lane in ~5 ml of RB for 15 min in a small container whilst gently shaking.

16.3 Discard RB and soak slice twice in ~5 ml of EB for 10 min.
Gel solutions can be made up during this time if 10% APS and TEMED are omitted.

16.4 Ensure that glass plates and Mini-Protean Tetra Vertical Electrophoresis Cell system are clean and free from dust.

16.5 Assemble glass plates with a 0.75-mm spacer as per Biorad protocol, sandwiching the gel slice in the region of the plates where the stacking gel is to be poured (Fig. 2). There should be a few mm between the top of the gel slice and the top of the plates so that the gel slice can be completely sealed into stacking gel. Note the orientation of the gel slice. For reference, a single tooth of a comb can be inserted to create a well for a molecular weight marker.

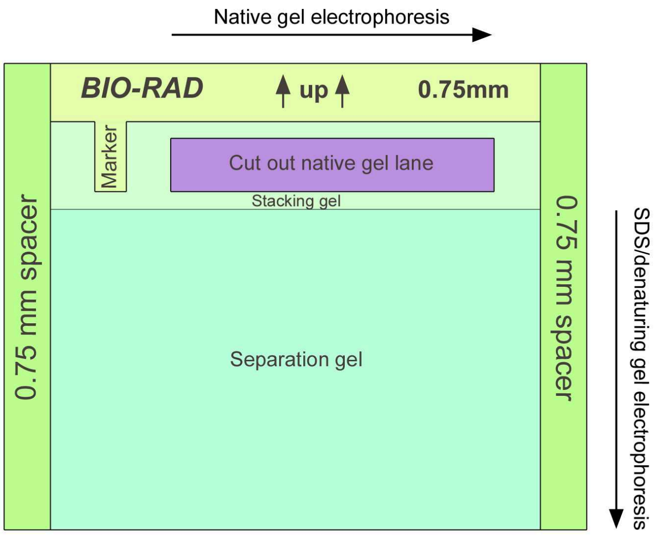

Figure 2 Assembly for casting second dimension gel with the Mini-Protean Tetra Vertical Electrophoresis Cell system (Biorad Laboratories).

16.6 Pour the separating gel after mixing in the 10% APS, and TEMED (Table 4). Enough space (~3 mm) should be left between the top of the separating gel and the gel slice for a stacking gel.

16.7 Overlay the separating gel with ~200 µl of water-saturated butanol and leave to polymerise.

16.8 When the gel has polymerised, remove the butanol and the layer of water from the top of the separating gel by upturning it over a sink and mop up the excess with thick filter paper.

16.9 Pour the stacking gel (Table 5), ensuring the gel slice is covered, and add a comb to create a well for a molecular weight marker. Stacking gels often seem to either leak or reduce in volume substantially at this point, so it is advisable to keep an eye on the level of the gel solution until it has polymerised.

16.10 Once the stacking gel has polymerised, leave the gels for 30 min before running.

16.11 Assemble electrophoresis cell as directed by Biorad ensuring there are no leaks.

16.12 Pour PB into inner and outer compartments of cell.

16.13 Load marker lane.

16.14 Run gel(s) at 100 V until the front from gel slice is well into stacking gel. Blue dye from the first dimension should still be slightly visible in gel slice and will concentrate substantially as it runs into the stacking gel.

16.15 Turn up the voltage to 200 V and run at constant a voltage until the blue front reaches bottom of gel.

16.16 Electro-blot and probe gel(s) for western blot analysis as directed in next section.

Western blotting and probing with antibodies

17 **Western blotting**

This protocol describes the electro-blotting of one-dimensional native gels or two-dimensional native/denaturing gels with the Mini Trans-blot Module from Biorad Laboratories.

Components

PVDF Transfer Membrane (0.2 µm)
- Thermo Fisher Scientific, Cat. No.: 88520.
- One piece per gel, cut slightly larger than the gel.

Methanol
- Store at room temperature.

Transfer buffer (TB): 25 mM Tris, 192 mM glycine, 20% methanol
- Dissolve 36.04 g of glycine and 7.57 g of Tris in 2 L of ddH_2O, then add 500 ml of methanol and mix.
- Store at 4°C.

Whatman 3MM Chromatography Paper

- Cytiva, Cat. No.: 3030-917.
- Two pieces per gel, cut slightly larger than the gel.

18 **Protocol for electro-blotting**

18.1 Write an identification code with a ballpoint pen on the PVDF blot.
Blot proteins on the side with the identification code.

18.2 Wet PVDF membrane in a small amount methanol before equilibrating for >5 min in TB.

18.3 Carefully remove the gel from plates: Place a razor blade between the glass plates to separate the glass plates. Then cut with the razor blade along the spacers to ensure that the gel does not rip when it is removed. Lift the gel from the bottom with tweezers/gloved fingertips.

18.4 Prepare the gel and membrane sandwich in a large container as shown in Fig 3, submerged in TB.

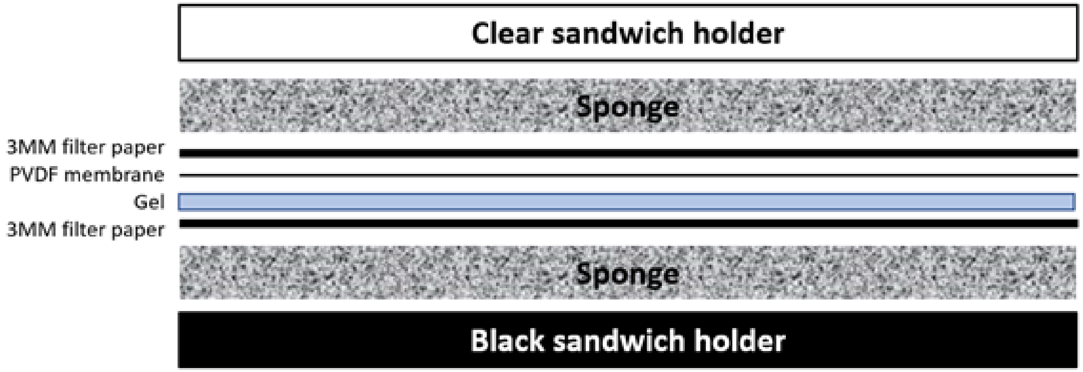

Figure 3 Sandwich for electro-blotting of gel onto PVDF membrane with the Mini Trans-blot Module (Biorad Laboratories).

18.5 Electro-blot at 100 V for 75 min in TB in Biorad tank as directed by the manufacturer with an ice pack and magnetic stirrer.

18.6 Remove membrane and allow to air-dry without washing if it is not to be used immediately.

The Serva Blue dye needs to be washed off the blotting membrane of blue-native gels before further use. This is done by rinsing the membrane in methanol 3 times for ~60 sec, after the membrane has air-dried completely. Wet membranes can be washed directly in methanol.

After methanol rinses, equilibrate blot for ~5 min in PBS.

19 **Probing of blot with antibodies and chemiluminescent development**

Components

Phosphate-buffered saline (PBS)
- 100 ml of 10x Dulbecco's Phosphate Buffered Saline (Gibco, Cat. No.: 14200-067) diluted with ddH_2O to 1 L.
- Store for <1 week at room temperature.

Blocking buffer
- 10 g of Skim Milk Powder (Millipore, Cat. No.: 70166-500G) in 100 ml of 1x PBS.
- Stir for 10 min to fully dissolve.
- Store for <2 d at 4°C.

Phosphate-buffered saline, 0.3% (v/v) Tween-20 (PBST)
- Pipette 3 ml of Tween-20 into 1 L of 1x PBS. (Use wide-bore pipette as Tween-20 is quite viscous).
- Shake bottle to dissolve Tween-20.
- Store for <1 week at room temperature.

Antibody incubation buffer
- Mix equal volumes of blocking buffer and PBST.
- Use fresh.

50-ml Tubes
- 50-ml screw top tubes, 114×28 mm; Sarstedt, Cat. No.: 62.547.254
- For antibody incubation of western blots.

Primary antibodies

Table 6 Suggestion of primary antibodies to detect each of the five oxidative phosphorylation complexes

Complex	Subunit	Supplier	Catalogue number	Suggested dilution
Complex I	NDUFB6	Abcam	ab110244	750^{-1}
Complex I	NDUFS3	Abcam	ab14711	500^{-1}
Complex II	SDHA	Abcam	ab14715	6000^{-1}
Complex II	SDHB	Abcam	ab14714	3000^{-1}
Complex III	UQCRC1	Abcam	ab110252	3000^{-1}
Complex III	UQCRC2	Abcam	ab14745	3000^{-1}
Complex IV	MTCO1	Abcam	ab14705	6000^{-1}
Complex IV	MTCO2	Abcam	ab110258	6000^{-1}
Complex V	ATP5A	Abcam	ab14748	12000^{-1}
Complex V	MTATP8	Proteintech	26723-1-AP	6000^{-1}

Secondary antibodies

- Anti-mouse IgG (H+L), HRP Conjugate; Promega, Cat. No.: W4021; dilute 6000^{-1}
- Anti-rabbit IgG (H+L), HRP Conjugate; Promega, Cat. No.: W4011; dilute 6000^{-1}

- **Clarity Western ECL Substrate**, Biorad Laboratories, Cat. No.: 1705061

- **Two transparent overhead sheets** (or cling film).

- **Chemidoc Imaging System**, Biorad Laboratories

20 **Protocol**

20.1 If the blot has been air-dried, wet blot in a small container with methanol and equilibrate in PBS.

20.2 Block the blot in a container in ~30 ml of blocking buffer for 1 h, whist shaking gently.

20.3 Rinse twice with PBST.

20.4 Put blot in a 50-ml tube with the blotted side towards the inside of the tube.
Add primary antibody (Table 6) diluted in 3 ml of antibody incubation buffer to the tube and place the tube on a bottle roller.
Incubate overnight at 4°C.

20.5 Wash the blot in the 50-ml tube three times for 10 min with ~15 ml of PBST on a bottle roller.

20.6 Incubate the blot in the 50-ml tube with 0.5 µl of the appropriate secondary antibody (HRP conjugate) diluted in 3 ml of antibody incubation buffer on a bottle roller for 1 h.

20.7 Wash the blot in the 50-ml tube three times for 10 min with ~15 ml of PBST on a bottle roller.

20.8 Transfer the blot to a small container and wash once for 5 min in ~30 ml of PBST.

20.9 Pipette 1 ml of Clarity Western ECL Substrate Peroxidase Solution and 1 ml of Clarity Western ECL Substrate Luminol/enhancer Solution into a new 50-ml tube.
Add the blot to the 50-ml tube with the blotted side towards the inside of the tube.
Incubate for 5 min on a bottle roller.

20.10 Remove the blot from the tube and place between two transparent overhead sheets with the blotted side facing upwards.
Capture images with the Chemidoc Imaging System.

20.11 Quantify signals with the software of the Chemidoc Imaging System.

Protocol references

Preparation of blue-native gels
Schägger, H. (1995). Quantification of oxidative phosphorylation enzymes after blue native electrophoresis and two-dimensional resolution: Normal complex I protein amounts in Parkinson's disease conflict with reduced catalytic activities. *Electrophoresis*, **16**(1):763-770. https://doi.org/10.1002/elps.11501601125

Sample preparation for analysis of individual oxidative phosphorylation complexes
Schägger, H., Bentlage, H., Ruitenbeek, W., Pfeiffer, K., Rotter, S., Rother, C., Böttcher-Purkl, A. & Lodemann, E. (1996). Electrophoretic separation of multiprotein complexes from blood platelets and cell lines: Technique for the analysis of diseases with defects in oxidative phosphorylation. *Electrophoresis*, **17**(4): 709-714. https://doi.org/10.1002/elps.1150170415

In-gel activity staining
Zerbetto, E., Vergani, L. & Dabbeni-Sala, F. (1997). Quantification of muscle mitochondrial oxidative phosphorylation enzymes via histochemical staining of blue native polyacrylamide gels. *Electrophoresis*, **18**(11): 2059-2064. https://doi.org/10.1002/elps.1150181131

Sample preparation for analysis of respiratory supercomplexes
Schägger, H. (2000). Supercomplexes in the respiratory chains of yeast and mammalian mitochondria. *The EMBO Journal*, 19(8): 1777-1783. https://doi.org/10.1093/emboj/19.8.1777

Extensive protocol for blue-native gel electrophoresis
Wittig, I., Braun, H.-P. & Schägger, H. (2006). Blue native PAGE. *Nature Protocols*, **1**(1): 418-428. https://doi.org/10.1038/nprot.2006.62

Clear-native gel electrophoresis and in-gel activity staining
Wittig, I., Karas, M. & Schägger, H. (2007). High resolution clear native electrophoresis for in-gel functional assays and fluorescence studies of membrane protein complexes. *Molecular and Cellular Proteomics*, **6**(7): 1215-1225. https://doi.org/10.1074/mcp.M700076-MCP200

Enhancement of Complex V in-gel activity staining
Suhai, T., Heidrich, N. G., Dencher, N. A. & Seelert, H. (2009). Highly sensitive detection of ATPase activity in native gels. *Electrophoresis*, **30**(20): 3622-3625. https://doi.org/10.1002/elps.200900114

Acknowledgements

We would like to acknowledge Dr Sîon L. Williams who developed the original method in our lab.
