# Supplementary material for: Validation of blue- and clear-native polyacrylamide gel electrophoresis protocols to characterize mitochondrial oxidative phosphorylation complexes
Source: PLoS One. 2025 Sep 18;20(9):e0332065. doi: 10.1371/journal.pone.0332065 (PMC12445495; doi:10.1371/journal.pone.0332065)
Supplement: S1 Table — (PDF) [file pone.0332065.s003.pdf]

S1 Table. siRNA species

Gene	siRNA	Catalogue number
NDUFV1	Hs_NDUFV1_1	Qiagen, S100094507
NDUFV1	Hs_NDUFV1_2	Qiagen, S100094514
NDUFV1	Hs_NDUFV1_3	Qiagen, S100094521
NDUFV1	Hs_NDUFV1_5	Qiagen, S103082478
NDUFV2	Hs_NDUFV2_1	Qiagen, S100129122
NDUFV2	Hs_NDUFV2_5	Qiagen, S103030657
NDUFV2	Hs_NDUFV2_6	Qiagen, S103097290
NDUFV2	Hs_NDUFV2_7	Qiagen, S103107881
NDUFS2	Hs_NDUFS2_1	Qiagen, S100657027
NDUFS2	Hs_NDUFS2_5	Qiagen, S103211705
NDUFS2	Hs_NDUFS2_6	Qiagen, S104243939
NDUFS2	Hs_NDUFS2_7	Qiagen, S104353538
NDUFS4	Hs_NDUFS4_1	Qiagen, S100038535
NDUFS4	Hs_NDUFS4_2	Qiagen, S100038542
NDUFS4	Hs_NDUFS4_3	Qiagen, S100038549
NDUFS4	Hs_NDUFS4_5	Qiagen, S103029201
