# Supplementary material for: Validation of blue- and clear-native polyacrylamide gel electrophoresis protocols to characterize mitochondrial oxidative phosphorylation complexes
Source: PLoS One. 2025 Sep 18;20(9):e0332065. doi: 10.1371/journal.pone.0332065 (PMC12445495; doi:10.1371/journal.pone.0332065)
Supplement: S2 Table — (PDF) [file pone.0332065.s004.pdf]

S2 Table. Primary antibodies

OXPHOS Complex	Subunit	Supplier	Catalogue number
Complex I	NDUFB6	Abcam	ab110244
Complex I	NDUFA9	Abcam	ab14713
Complex I	NDUFS2	Abcam	ab110249
Complex I	NDUFS4	Abcam	ab87399
Complex I	NDUFV1	ProteinTech	11238-1-AP
Complex I	NDUFV2	ProteinTech	15301-1-AP
Complex I	MTND1	Kind gift from Dr A. Lombès	
Complex II	SDHA	Abcam	ab14715
Complex III	UQCRC2	Abcam	ab14745
Complex IV	MTCO1	Abcam	ab14705
Complex IV	MTCO2	Abcam	ab110258
Complex V	ATP5A	Abcam	ab14748
Complex V	MTATP8	ProteinTech	26723-1-AP
