# Supplementary material for: Validation of blue- and clear-native polyacrylamide gel electrophoresis protocols to characterize mitochondrial oxidative phosphorylation complexes
Source: PLoS One. 2025 Sep 18;20(9):e0332065. doi: 10.1371/journal.pone.0332065 (PMC12445495; doi:10.1371/journal.pone.0332065)
Supplement: S3 Table — (PDF) [file pone.0332065.s005.pdf]

S3 Table. Secondary antibodies

Antibody	Supplier	Catalogue number
Anti-Rabbit IgG (H+L), HRP conjugate	Dako	PO448
Anti-Mouse IgG (H+L), HRP conjugate	Dako	PO447
Anti-Mouse IgG (H+L), HRP conjugate	Promega	W4021
