# Supplementary material for: Validation of blue- and clear-native polyacrylamide gel electrophoresis protocols to characterize mitochondrial oxidative phosphorylation complexes
Source: PLoS One. 2025 Sep 18;20(9):e0332065. doi: 10.1371/journal.pone.0332065 (PMC12445495; doi:10.1371/journal.pone.0332065)
Supplement: S1 Fig — (PDF) [file pone.0332065.s006.pdf]

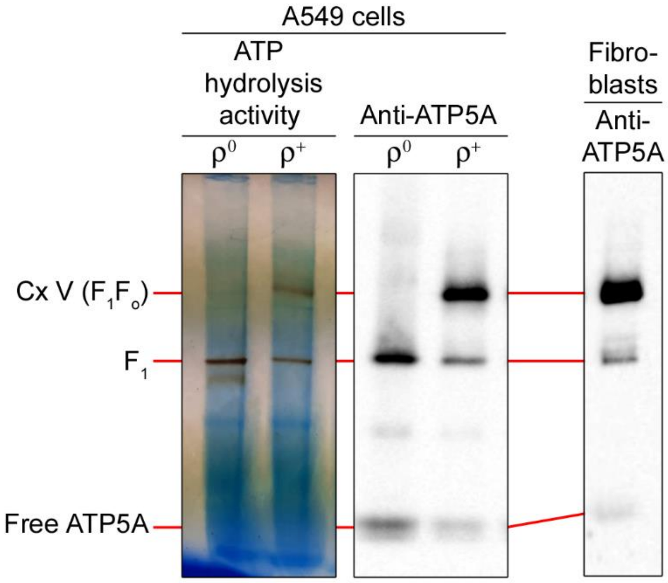

S1 Fig. In-gel activity and western blot detection of Complex V after BN-PAGE. A549 (ρ^+), A549 ρ^0 (ρ^0) and fibroblast *n*-dodecyl-β-D-maltoside extracts (10 μg/lane) were resolved by 3–12% BN-PAGE, followed by in-gel ATP hydrolysis activity staining or western blot analysis with Complex V F_1-domain subunit ATP5A antibodies. Migration of Complex V (Cx V), the F_1-part and free, unassembled ATP5A is indicated. Note that the F_1-part is still assembled in the sample from A549 ρ^0 cells that are devoid of mtDNA. The F_1-part retains its ATP hydrolysis activity. Also note that in addition to holo-Complex V, the F_1-part is detected the samples from A549 cells and fibroblasts, suggesting that Complex V is partly dissociated.
