# Supplementary material for: Validation of blue- and clear-native polyacrylamide gel electrophoresis protocols to characterize mitochondrial oxidative phosphorylation complexes
Source: PLoS One. 2025 Sep 18;20(9):e0332065. doi: 10.1371/journal.pone.0332065 (PMC12445495; doi:10.1371/journal.pone.0332065)
Supplement: S2 Fig — (PDF) [file pone.0332065.s007.pdf]

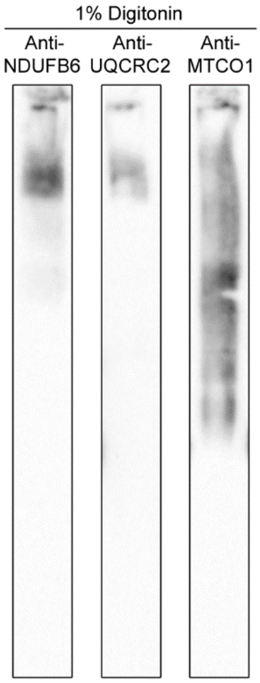

S2 Fig. Western blot analysis of a 1% digitonin extract from HEK293T cells. A HEK293T cell suspension was extracted with 1% digitonin, followed by 3–10% BN-PAGE (10 μg protein extract/lane) and western blot analysis with indicated antibodies.
