# Supplementary material for: Validation of blue- and clear-native polyacrylamide gel electrophoresis protocols to characterize mitochondrial oxidative phosphorylation complexes
Source: PLoS One. 2025 Sep 18;20(9):e0332065. doi: 10.1371/journal.pone.0332065 (PMC12445495; doi:10.1371/journal.pone.0332065)
Supplement: Attachment — Submitted filename: Response to reviewers.pdf [file pone.0332065.s009.pdf]

DEPARTMENT OF CLINICAL AND MOVEMENT NEUROSCIENCES
QUEEN SQUARE INSTITUTE OF NEUROLOGY
UNIVERSITY COLLEGE LONDON
Rowland Hill Street
London NW3 2PF
United Kingdom

Dr Peng Gao, Editor – *PLOS ONE*

12 August 2025

Dear Dr Gao,

Re: **Validation of blue- and clear-native polyacrylamide gel electrophoresis protocols to characterize mitochondrial oxidative phosphorylation complexes** (re-submission)

We thank you for your time and consideration concerning our manuscript. We thank you for sending us the constructive comments from the two reviewers and giving us the opportunity to address their concerns. We hope that our revised manuscript is now acceptable for publication in *PLOS ONE*.

As suggested, regarding the experiments involving animals, we have now included details on (1) methods of sacrifice, (2) methods of anesthesia and (3) efforts to alleviate suffering.

Reviewer #1:
The study presents a comprehensive validation of BN-PAGE and CN-PAGE protocols for characterizing OXPHOS complexes, with rigorous experimental design and robust supporting data. The following points should be addressed:

We thank Reviewer 1 for the time and consideration to review our manuscript.

1. The authors should provide an explanation for the observed enhanced Complex IV staining in liver mitochondria relative to cultured cells. Given the liver's well-established high mitochondrial density and elevated metabolic activity, these inherent biological characteristics likely contribute to the differential staining outcomes. Clarification of this relationship would facilitate proper interpretation of the tissue-specific results.

We have now provided an explanation for the observed enhanced Complex IV staining in liver mitochondria relative to cultured cells in the Discussion, as suggested by Reviewer 1.

2. For the technical limitations regarding Complex III/IV staining, additional discussion of potential underlying factors (e.g., enzyme stability or detergent compatibility) and attempted optimization strategies would strengthen the methodology section.

We explain in the text that in-gel Complex IV activity staining is not particularly sensitive and that differences in OXPHOS enzyme content in the liver mitochondrial samples relative to the whole cell extract samples are likely to explain the inability to detect Complex IV activity by in-gel staining. We mention in the text that also others [22,42] have been unable to stain gels with cultured cell fractions for Complex IV activity in a conclusive manner.

To our knowledge, only one paper has claimed to achieve in-gel Complex III activity staining [21]. The authors used a bovine heart mitochondrial sample and incubated the gel in 0.5 mg/ml of diaminobenzidine (DAB) in 50 mM sodium phosphate (pH 7.2). No explanation is given why this incubation should result in a specific Complex III staining. Possibly, a peroxidase comigrating with Complex III oxidizes DAB, producing a brown indamine polymer. We have attempted to stain gels for Complex III with DAB but this did not result in any staining.

Tel: +44 (0)20 8016 8148 E-mail j.taanman@ucl.ac.uk

3. The wide range of protein loading quantities (5-30 µg) warrants clearer justification, especially whether this reflects biological sample differences or stain-specific sensitivity requirements.

We have now added a paragraph to the Discussion, which discusses protein loading quantities.

4. The protocol's innovative aspects - particularly the streamlined extraction and enhanced Complex V detection - should be more prominently featured with direct comparisons to conventional methods.

We have now mentioned the innovative streamlined extraction and enhanced Complex V detection in the first paragraph of the Discussion.

5. Please discuss the similarities, differences, and innovations of this study in comparison to the referenced work (DOI: 10.3791/59294), particularly focusing on methodological advancements and biological implications.

We have now included the paper by Cuillerier & Burelle (DOI: 10.3791/59294) in our References ([14]) and refer to it in the manuscript twice.

6. Finally, a brief discussion of potential clinical applications, especially for mitochondrial disorder diagnostics, and sample-specific adaptation considerations would enhance the paper's translational impact.

We have now added an extra paragraph at the end of the Discussion to discuss the potential applications of this protocol in diagnosing mitochondrial diseases and studying the effects of drugs on the OXPHOS system.

Reviewer #2: This article validates a blue and transparent natural polyacrylamide gel electrophoresis (BN-PAGE and CN-PAGE) protocol for characterizing mitochondrial oxidative phosphorylation complexes. The study clearly describes the experimental protocol and provides sufficient experimental data to support its conclusions. This research has potential practical value as it simplifies the analysis process of mitochondrial oxidative phosphorylation complexes and enhances the sensitivity of certain enzyme activity staining.
Here are two suggestions:

We thank Reviewer 2 for the time and consideration to review our manuscript.

1. The results section can be more concise and clear. For example, the description of the Western blot results can be simplified, with a focus on highlighting the key findings.

As this is a “Lab Protocol Article”, we feel that a thorough description of the results is needed for those who are new to the technique.

2. The discussion section can also explore the application prospects of this protocol in future research. For instance, it can discuss the potential applications of this protocol in diagnosing mitochondrial diseases and studying the effects of drugs on the OXPHOS system.

We have now added an extra paragraph at the end of the Discussion to discuss the potential applications of this protocol in diagnosing mitochondrial diseases and studying the effects of drugs on the OXPHOS system.

On behalf of all authors.

Yours faithfully,

Jan-Willem Taanman, PhD
Associate Professor of Clinical Neurosciences

Tel: +44 (0)20 8016 8148 E-mail j.taanman@ucl.ac.uk
